# The prevalence and impacts heavy menstrual bleeding on anemia, fatigue and quality of life in women of reproductive age

**DOI:** 10.12669/pjms.35.2.644

**Published:** 2019

**Authors:** Semra Kocaoz, Rabiye Cirpan, Arife Zuhal Degirmencioglu

**Affiliations:** 1 *Dr. Semra Kocaoz, PhD, Associate Professor, Nigde Zubeyde Hanim School of Health, Nursing Department, Department of Obstetrics and Gynecology Nursing, Nigde Omer Halisdemir University, Nigde, Turkey*; 2 *Dr. Rabiye Cirpan, PhD, Assistant Professor, Nigde Zubeyde Hanim School of Health, Nursing Department, Department of Internal Medicine Nursing, Nigde Omer Halisdemir University, Nigde, Turkey*; 3 *Dr. Arife Zuhal Degirmencioglu, MD, Medical Faculty, Medical Sciences Department, Department of Internal Medicine, Nigde Omer Halisdemir University, Nigde, Turkey*

**Keywords:** Anemia, Fatigue, Menstruation, Quality of life, Reproductive Period

## Abstract

**Objectives:**

To investigate the prevalence and impacts of heavy menstrual bleeding (HMB) on anemia, fatigue, and the quality of life (QoL) in women of reproductive age.

**Methods:**

This study was conducted among 306 women of reproductive age who presented at the internal medicine outpatient departments of the training and research hospital of a university. The data of the study were collected by the “Data collection form”, “SF-36 Quality of Life Scale (SF-36 QoLS)” and “Brief Fatigue Inventory (BFI)”.

**Results:**

The prevalence of HMB in women of reproductive age was 37.9%. The ferritin level and physical functions were found to decrease significantly as the duration of menstruation increased (*p*<0.05). Besides, a positive but very weak relationship was found between the menstruation duration and the subdimensions of the global BFI and the general health perception subscale of the SF-36 QoLS (*p*<0.05).

**Conclusion:**

It was determined that HMB is common and has negative effects on anemia, fatigue and some subdimensions of the QoL. Regular screening for HMB that may not be expressed by many women may therefore be useful in preventing and resolving the health problems that it will cause.

## INTRODUCTION

HMB has been objectively described as “the loss of 80 ml or more blood during every menstrual cycle” in the 1960s.^1^ This definition has been changed by the NICE in the United Kingdom in 2007 to “excessive menstrual blood loss that physically, emotionally, socially and financially affects the QoL of women and can be seen by itself or with other symptoms” to include the above definition.^2^ Reported HMB prevalence in women varies between 27.2% and 54.0%^3^-^9^, making it a common disorder. The cause of HBM can be fibroids and polyps, adenomyosis, irregular ovulation, bleeding disorders, drugs, cancer and other etiologic factors.^10^ HMB has been shown to have negative effects on the psychological state, energy, work productivity, social relationships, family life and sexual functions of women.^4^,^11^,^12^ Besides, the excessive blood loss during the menstrual period can cause physical health problems such as iron deficiency anemia (IDA) and fatigue.^6^,^13^,^14^

Fatigue and IDA developing due to excessive menstrual bleeding affects the QoL of women negatively.^4^ Besides, HMB itself is also reported to have the potential to cause problems.^4^,^11^,^12^ It was emphasized in a study that the woman’s experience with blood loss and the effect on her life should be identified when planning the treatment of abnormal uterine bleeding.^11^ We therefore aimed to find the prevalence of HMB in women of reproductive age and to evaluate the effects of this problem on anemia, fatigue and the QoL.

## METHODS

This descriptive and sectional study was conducted with women in the age group of 15-49 years who presented to the internal medicine outpatient departments of the training and research hospital of a university between 15.01-02.04.2018 to be examined. It was not possible to determine how many women aged 15-49 years had presented to the outpatient departments for examination from the automation system of the study hospital so we used the sample size from unknown population when selecting the sample. Using the sample size from unknown population formula with a 95% confidence interval and p-value of 0.272^3^, the minimum number of women required in the sample was calculated as 304.

Study inclusion criteria were women in the age group of 15-49 years who consented to participate in the study and could understand and answer the questions besides having regular menstrual periods.

The study was completed with 306 women who met the above criteria. After the necessary permissions were obtained, data collection tools were administered to the women with the face-to-face interview method by the investigators and a trained pollster at the internal medicine outpatient department of the hospital where the study was conducted.

The data were collected with the “Data Collection Form” created by the investigators after evaluating the relevant literature^3^,^7^,^13^,^15^, the “SF-36 QoLS and the BFI. The data collection form included a total of 22 questions regarding the women’s socio-demographic characteristics, menstrual features and other risk factors in addition to the hemoglobin and serum ferritin level. The presence of HMB was defined as menstruation continuing for more than 7 days^16^ and according to the criteria used by Fraser et al.^3^ in their study.

The SF-36 QoLS was developed in 1987 by Ware to be used in the investigation of the health-related QoL. This scale contains 36 items and includes two main titles and eight subdimensions. This scale has positive scoring and the QoL of the individual increases as the score obtained in each health area increases.^17^

The BFI was developed by the MD Anderson Cancer Center in order to identify the level of fatigue. This inventory includes the general fatigue level and the level of interference with daily activities within the past 24 hours. A score of “0” from the BFI shows that fatigue does not affect the individual, “1-3” a low level, “4-6” a moderate level, “7-9” a high level, and “10” the highest level.^18^

This study was started after obtaining approval from the Nigde Omer Halisdemir University Ethics Committee and written permission from hospital where the study was conducted. Before administering the data collection tools, the aim of the study was explained to women aged 15-49 years and they were asked to voluntarily participate in the study. Verbal and written informed consent was obtained from the women who accepted to participate.

### Statistical Analysis

The study data were evaluated by using the SPSS IBM (24.0) software. Descriptive statistics, t-test and Mann-Whitney U-test were used for data analysis. The Spearman Correlation test was used in the evaluation of the relationship between menstruation duration and hemoglobin and serum ferritin levels, fatigue and QoL. A *p* value of <0.05 was considered significant.

## RESULTS

The mean age of the women and at menarche (years) was 30.8±9.7 and 13.4±1.4 respectively. The menstruation duration was 8 or more days in 15.4% of the women. We found that 54.9% were using more than 12 pads during one menstrual period and 13.1% were experiencing intense menstrual bleeding of 4 days or longer. Large clot passage during menstruation was present in 66.3%. Besides, 27.1% expressed that they used double pads, 53.3% often replaced the pad to avoid blood overflow, and 8.2% had intermittent bleeding. Using our study criteria, the prevalence of HMB was 37.9% in the female population. We found that 24.1% of the women perceived the bleeding they experienced during menstruation as heavy or very heavy (Table-I).

**Table-I:** Menstrual features of women.

Menstruation Related Features	n	%
*Age at menarche* (X̄*±SD=13.4±1.4) (Min-Max=9-18)*		
≤11	20	6.5
12-14 years	223	72.9
≥ 15 years	63	20.6
*Duration of menstruation* (X̄*±SD=6.15±1.8) (Min-Max=1-12)*		
≤ 4 days	45	14.7
5-7 days	214	69.9
≥8 days	47	15.4
*Menstrual cycle duration* (X̄*±SD=26.7±6.3) (Min-Max=10-60)*		
<21 days	40	13.1
21-35 days	253	82.7
>35 days	13	4.2
*Number of pads used in one cycle* (X̄*±SD=13.7±9.6) (Min-Max=1-100)*		
≤11 pads	138	45.1
≥12 pads	168	54.9
*The number of days when menstruation is heavy*		
Never	7	2.2
1 day	21	6.9
2 days	119	38.9
3 days	119	38.9
≥ 4 days	40	13.1
*Large clot passage during menstruation*		
Yes	203	66.3
No	103	33.7
*Use of double pads during menstruation*		
Yes	83	27.1
No	223	72.9
*Frequent replacement of pads for menstrual bleeding to prevent overflow*		
Yes	163	53.3
No	143	46.7
*Intermittent bleeding*		
Yes	25	8.2
No	281	91.8
*Presence of HMB meeting criteria*		
Yes	116	37.9
No	190	62.1
*Severity of menstrual bleeding as perceived by women*		
Mild	47	15.4
Moderate	185	60.5
Heavy	50	16.3
Very heavy	24	7.8

Anemia diagnosis was present in 63.4% of the women in our study; 33.0% reported that they had used an iron preparation in the last three months and 16.7% were still using it. The mean hemoglobin amount and serum ferritin levels in women of reproductive age were 13.41±1.3 g/dL and 32.76±8.0 ng/mL, respectively. Besides, 14.0% of the women had a hemoglobin level of <12 g/dL and 17.3% had a serum ferritin level under 7 ng/mL.

The median hemoglobin and ferritin levels of the HMB group were found to be statistically significantly lower than the group without HMB (*p<*0.05). A statistically significant relationship was found between HMB presence and the median general fatigue and interference with daily activities scores (*p<*0.05). The QoL was found to be higher in the physical functioning and role limitation (physical and emotional) subdimensions but lower in the bodily pain subdimension of the SF-36 QoLS in women without HMB than those with HMB (*p<*0.05). No statistically significant relationship was found between HMB presence and the other subdimensions of SF-36 QoLS (*p>0.05*) (Table-II).

**Table-II:** Presence of HMB according to the Hemoglobin and Ferritin Levels, and the BFI and SF-36 QoLS Subdimension Scores

	Presence of HMB				

Hemoglobin and Ferritin Levels	Yes		No		Test/p-value

	x̄±SD	Median	x̄±SD	Median	
Hemoglobin*	13.05±1.4	13.25	13.64±1.2	13.85	Z=-3.896 p<0.0001
Ferritin*	23.36±4.0	11.45	38.50±9.7	19.65	Z=-3.999 p<0.0001
*Brief Fatigue Level*					
General Fatigue Level*	6.19±2.3	6.50	5.07±2.6	5.33	Z=-3.564 p<0.0001
Interference with Daily Activities*	4.20±2.6	4.33	3.55±2.8	3.08	Z=-2.258 p=0.024
*SF-36 QoLS Subdimensions*					
SF-36 Physical Functioning*	73.58±2.5	82.50	80.24±2.1	85.00	Z=-2.438 p=0.015
SF-36 Role Limitation-Physical*	51.94±4.0	50.00	64.21±1.2	75.00	Z=-2.720 p=0.007
SF-36 Bodily Pain*	41.55±2.4	40.00	35.58±2.5	30.00	Z=-2.040 p=0.041
SF-36 General Health Perception*	52.76±1.3	50.00	52.13±1.2	50.00	Z=-0.567 p=0.571
SF-36 Energy/ Vitality*	49.74±1.2	50.00	48.71±1.4	50.00	Z=-1.035 p=0.301
SF-36 Mental Health*	46.86±1.3	48.00	48.51±1.4	48.00	Z=-1.149 p=0.251
SF-36 Role Limitation-Emotional*	51.44±4.4	66.67	65.09±4.1	83.33	Z=-2.695 p=0.007
SF-36 Social Functioning*	45.80±1.5	50.00	47.83±1.5	50.00	Z=-1.190 p=0.234
SF-36 Physical Component Summary*	36.43±6.8	37.03	37.93±6.8	38.52	Z=-1.767 p=0.077
SF-36 Mental Component Summary**	43.09±6.4	43.39	43.70±5.6	43.51	t=-0.878 p=0.266

* Mann-Whitney U test,

** Independent Samples t test

According to the Spearman’s correlation analysis conducted, the ferritin level slightly decreased and the fatigue levels increased in women as the menstruation level increased (*p<*0.05). We found a weak negative significant relationship between the menstruation duration and the physical functioning subdimension of SF-36 QoLS and a weak positive significant relationship between the menstruation duration and the general health perception subdimension (*p<*0.05). No statistically significant relationship was found between the menstruation duration and the other subdimensions of SF-36 QoLS than physical functionality and general health perception (*p>0.05*) (Table-III).

**Table-III:** The Relationship between the Hemoglobin and Ferritin Levels, SF-36 QoLS Subdimensions, Components of the BFI, and Menstruation Duration.

	Menstruation Duration	

Laboratory Values	r	p
Hemoglobin	-0.100	0.080
Ferritin	-0.121	0.035
*SF-36 QoLS Subdimensions*		
SF-36 Physical Functioning	-0.144	0.011
SF-36 Role Limitation-Physical	-0.104	0.070
SF-36 Bodily Pain	0.089	0.121
SF-36 General Health Perception	0.156	0.006
SF-36 Energy/ Vitality	0.102	0.074
SF-36 Mental Health	0.042	0.466
SF-36 Role Limitation-Emotional	-0.044	0.444
SF-36 Social Functioning	-0.028	0.630
SF-36 Physical Component Summary	0.042	0.467
SF-36 Mental Component Summary	-0.085	0.139
*BFI*		
General Fatigue Level	0.157	0.006
Interference with Daily Activities	0.114	0.047

## DISCUSSION

HMB was found to be present in approximately 4 of 10 (37.9%) women of reproductive age in our study (Table-III). This rate was reported as 27.2% in women aged 16-57 years living in five European regions in a study on the prevalence of HMB using similar criteria.^3^ The prevalence of heavy menstruation was found to be 32% in the study conducted by Karlsson et al.^4^ among women aged 40-45 years in Sweden. This prevalence was found to be lower at 15.2% in a study conducted among women aged 15-45 years in Iran.^8^ There is no previous study on the prevalence of HMB in women of reproductive age in our country, but the prevalence of HMB was 21.8% in a study conducted on university students.^9^ The higher prevalence of HMB in our study compared to other studies^3^,^9^ on women in a similar age group could be due to the difference sample population and data collection method and also because the subjects presenting at the internal medicine outpatient departments for the follow-up and treatment of anemia that could be the result of heavy menstruation.

Despite the various definitions of HMB^1^,^5^,^16^, it is emphasized that the perception of this condition by women should also be investigated.^16^ The menstrual bleeding was thought to be heavy or very heavy in 24.1% of the women in our study (Table-I). An Australian study reported that 27.8% of the women aged 20-39 years perceived their menstrual bleeding as heavy or very heavy.^19^ Our finding is similar to that reported by Weisberg, McGeehan, Fraser.^19^

HMB can therefore cause a decrease in iron level and hemoglobin amount and lead to anemia in women if not treated.^20^Anemia was found to be present in two third of women who had HMB in the study of Fraser et al.^3^ The presence of HMB and the relationship between hemoglobin and serum ferritin in women were investigated according to the two parameters of the hemoglobin amount and serum ferritin level used for the detection of anemia in our study. The median hemoglobin amount and ferritin levels of women who were found to have HMB were significantly lower than those without heavy bleeding (*p<0*.0001). Our results are similar to those reported in the literature.^1^,^3^,^20^ Besides, the ferritin level was found to decrease mildly as the menstruation duration increased in our study. The lack of or weak correlation between the hemoglobin amount and ferritin level and menstruation duration in our study could be due to two thirds of the women in the sample having used iron preparations in the last three months.

HMB can cause fatigue in addition to iron deficiency in women.^6^,^13^,^14^ Fatigue (90.4%) was reported to be one of the main symptoms in women in the study conducted by Fraser et al.^3^ Similarly, Wang et al.^14^ found the fatigue to be one of the most common symptoms in young women with HMB in their study. The median general fatigue level (*p<*0.0001) and interference with daily activities (*p=*0.024) scores of women with HMB were found to be significantly higher than those without HMB in our study. Besides, a very weak positive relationship was found between the menstruation duration and the general fatigue level (*p*=0.006) and the level of interference with daily activities (*p*=0.047). Our results are consistent with the above-mentioned literature.^3^,^6^,^13^,^14^

HMB itself 4,11,12 and the anemia and fatigue developing due to the bleeding^4^ are reported to decrease the QoL in women. However, it is not clear how the symptoms caused by HBM affect the QoL in women.^21^ The QoL in the physical functioning and role limitation (physical and emotional) subdimensions of the SF-36 QoLS was found to be significantly low in presence of HBM in women in our study (*p<*0.05). Besides, the median physical and mental component summary scores of QoLS of SF-36 decreased in the presence of HMB, although not statistically significant. The QoL in all subdimensions of SF-36 QoLS was found to decrease in women with HMB compared to those with normal menstruation in the study of Karlsson et al.^4^ A statistically significant difference in all the subdimensions of SF-36 was found between the women with menorrhagia and the control group in a case control study conducted in our country.^15^

We also found a weak negative correlation between menstruation duration and the physical functioning subdimension of SF-36 QoLS and also a weak positive correlation between menstruation duration and the health perception subdimension in our study. However, no significant relationship was found between menstruation duration and the SF-36 QoLS physical and mental component summary scores. The limitation in physical and social/leisure time activities was found to increase as the daily blood loss increased in women in the study conducted by Lukes et al.^21^ De Souza et al.^22^ found no significant relationship between the menstruation amount and the SF-36 QoLS physical and mental component in their study. The lack of a significant relationship between any subdimension of the QoL and menstruation duration in our study could be due to the majority of the women using an iron preparation for anemia. This view is supported by our finding that the hemoglobin amount of women and their physical and mental component summary scores of QoLS in SF-36 increased significantly after treatment in the study of de Souza et al.^22^

## CONCLUSION

HMB is common in women and was found to decrease the hemoglobin and serum ferritin levels used in evaluating anemia, increase the fatigue level, and affect the physical and role limitation (physical and emotional) subdimensions of the QoL negatively in our study. We believe that the negative effects on anemia, fatigue and QoL caused by this problem can be prevented and treatment plans can be identified by querying HMB in all women of reproductive age who present for examination and by performing regular follow-ups. We recommend investigating the effects of HMB on anemia, fatigue and QoL by checking the use of iron preparations in future studies.
